# A mutant α1antitrypsin in complex with heat shock proteins as the primary antigen in type 1 diabetes in silico investigation

**DOI:** 10.1038/s41598-021-82730-2

**Published:** 2021-02-04

**Authors:** Paola Finotti, Andrea Pagetta

**Affiliations:** Department of Pharmaceutical and Pharmacological Sciences, University of Padua, Building “C”, Largo E. Meneghetti, 2, 35131 Padua, Italy

**Keywords:** Computational biology and bioinformatics, Immunology, Biomarkers, Diseases, Endocrinology, Medical research, Molecular medicine, Pathogenesis

## Abstract

Based on previous results demonstrating that complexes of a mutant α1-antitrypsin with the heat shock proteins (HSP)70 and glucose-regulated protein94 (Grp94) circulate in the blood of patients with type 1 diabetes, we raised the hypothesis that these complexes could represent the primary antigen capable of triggering the autoimmune reactions leading to overt diabetes. As a first approach to this issue, we searched whether A1AT and HSPs had a sequence similarity to major islet antigen proteins so as to identify among the similar sequences those with potential relevance for the pathogenesis of diabetes. A thorough in silico analysis was performed to establish the score of similarity of the human proteins: A1AT, pro-insulin (INS), GAD65, IAPP, IA-2, ICA69, Grp94, HSP70 and HSP60. The sequences of A1AT and HSPs with the highest score of similarity to the islet peptides reported in the literature as the main autoantigens in human diabetes were recorded. At variance with other HSPs, also including HSP90 and Grp78, Grp94 contained the highest number and the longest sequences with structural similarity to A1AT and to well-known immunogenic peptides/epitopes of INS, GAD65, and IA-2. The similarity of A1AT with Grp94 and that of Grp94 with INS also suggested a functional relationship among the proteins. Specific sequences were identified in A1AT, Grp94 and HSP70, with the highest score of cross-similarity to a pattern of eight different islet protein epitopes. The similarity also involved recently discovered autoantigens in type 1 diabetes such as a hybrid peptides of insulin and the defective ribosomal insulin gene product. The significant similarity displayed by specific sequences of Grp94 and A1AT to the islet peptides considered main antigens in human diabetes, is a strong indication for testing these sequences as new peptides of immunogenic relevance in diabetes.

## Introduction

Despite strenuous efforts to define the aetiology and pathogenic mechanism of type 1 diabetes (T1D), the precise cause of the disease remains unknown, and no specific cure is available^1^. None of the many factors identified thus far as causally related to diabetes, including the major islet cell auto-antigens, can explain the heterogeneous molecular and clinical features of the disease^2^. Thus, although antigen-specific immune-therapies have not met expectations^3–5^, the recognition that a wide range of different mechanisms concurs with beta cells destruction has prompted investigators to focus on broad-spectrum, non-antigen-specific, immune-based therapies^6^. Special focus has been placed on α1–antitrypsin (A1AT), the main circulating anti-proteinase inhibitor, which is used as a diabetes therapeutic agent for its broad anti-inflammatory and immune-modulatory properties unrelated to the specific proteinase inhibitory activity^7^. Although clinical trials of A1AT have not been successful^8^, the finding that A1AT is expressed in both islet^9^ and endothelial cells^10^ of the human pancreas, in addition to its protective effects on beta cells^11,12^ and islet transplantation^13^, corroborated the idea that A1AT actually plays a role in regulating islet cell homeostasis and glucose metabolism, confirmed by studies on genes related to the metabolic syndrome^14^.

The A1AT gene undergoes a high mutation rate: thus, there are many variants of the protein, with some also related to specific pathologies^15^. When mutations severely affect A1AT folding, so as to prevent protein secretion, the endoplasmic reticulum (ER) machinery employing specific heat shock proteins (HSPs) is set up to restore protein folding^16^. If this process fails, the mutated A1AT (mA1AT) is directed to the ER-associated-degradation pathway with the primary assistance of glucose-regulated protein94 (Grp94)^17^, the most represented ER HSP. The resulting intense ER stress that follows activation of the unfolded protein response (UPR) in A1AT-expressing cells is responsible for cell apoptosis^16^ and extracellular liberation of the protein content.

In previous works, we demonstrated that a variant, probably a mutated form of A1AT that is structurally and functionally distinct from normal A1AT (wA1AT), was present in the plasma of patients with T1D^18^ and linked to HSP70 in complexes with Grp94^19,20^. This finding was the unequivocal sign of an upstream defective folding mechanism of mA1AT eventually responsible for the intracellular events that result in the engagement and extracellular liberation of HSPs. Considering also that HSPs outside the cell are a potent immunogenic stimulus, and that HSPs are found in the plasma of diabetic subjects even years after diabetes onset^20^, we hypothesised that complexes of mA1AT with HSPs could represent a true, stable antigen in diabetes, that is responsible for initiating and maintaining autoimmune reactions. As a first investigative approach to this problem, we sought to determine whether A1AT and HSPs share a structural similarity to the pancreas islet proteins already identified as the main antigens in diabetes, and, in particular, whether this similarity indicates that the islet peptides are key players in autoreactivity and loss of tolerance in human diabetes. The comprehensive and thorough in silico analysis subsequently conducted on sequences of A1AT, HSPs and the main islet proteins provides preliminary information about the potential immunogenic implication of A1AT/HSP complexes in the pathogenesis of T1D.

## Methods

Human proteins were recovered through UniProtKB/Swiss-Prot (http://www.uniprot.org). Proteins (entry name with the accession number) were: alpha1 antitrypsin (A1AT, P01009-1); Pro-Insulin (INS, P01308-1); Islet Cell Autoantigen 1 (ICA69, Q05084); Glutamate Decarboxylase 2, GAD65 (DCE2, Q05329); Receptor-type Tyrosine-protein Phosphatase-like N, IA-2 (PTPRN, Q16849); Islet Amyloid Polypeptide (IAPP, P10997); Endoplasmin, Grp94 (ENPL, P14625); Heat Shock 70 kDa protein 1A (HS71A, P0DMV8); and 60 kDa HSP (CH60, P10809). Also retrieved were the sequences of human: Endoplasmic reticulum chaperone Bip (Grp78; P11021), Heat Shock Protein 90-alpha (HSP90AA1-1, P07900-1), Heat Shock Protein 40 (DNAJB1, P25685), Zinc transporter 8 (ZnT8, Q8IWV4), Glucose 6-phosphatase 2 (IGRP, Q9NQR9), Urocortin 3 (UCN3, Q969E3), Insulin gene enhancer protein isl-1 (ISL-1, P61371).

To detect sequence similarities, pairwise alignment of the proteins was performed using two progressive alignment methods, the Clustal Omega programme (UniProtKB) and the T-Coffee (Tree-based Consistency Objective Function for alignment Evaluation) package that combines different sequence-alignment programmes^21^. The two methods use different algorithms for sequence alignment; thus, different sequences can be detected as similar in the same protein. Although T-Coffee has proven to be superior to other methods, Clustal Omega was combined in any pairwise sequence alignment to detect as many similar sequences as possible, a condition not satisfied by a single method. The concordance of both methods in detecting sequence similarity added further relevance to the sequence similarity. The programmes chosen in the T-Coffee package to obtain consensus alignment were muscle msa (multiple sequence alignment) and T-Coffee msa, both of which start by computing pairwise alignments of the input sequences. The parameter setting included regular computation mode. Only sequences of at least eight consecutive amino acid residues without any gap and with the highest consistency score were considered in the computation.

**Figure 1:**
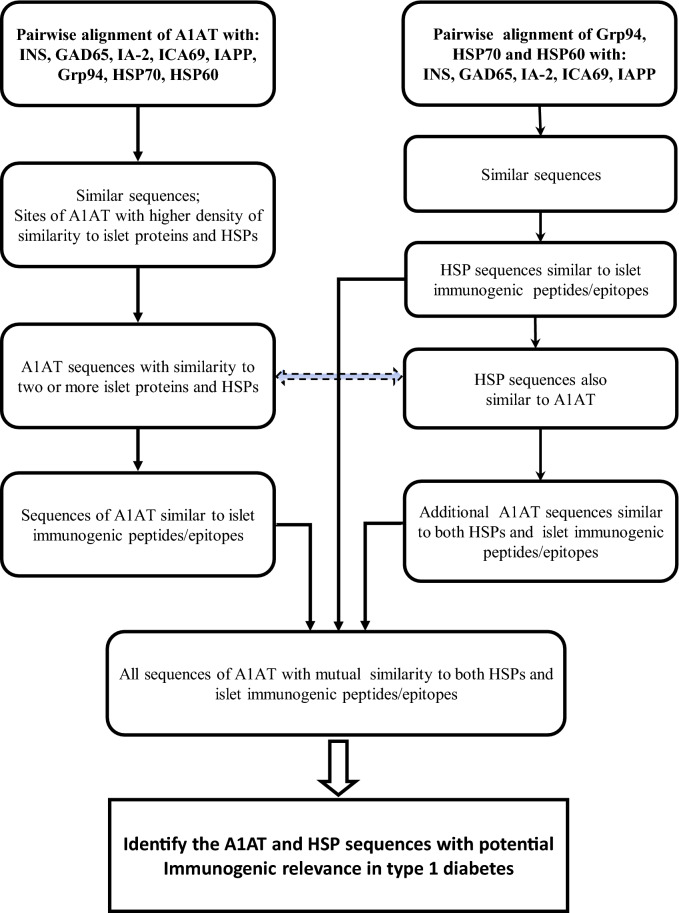
Flowchart of steps to identify similar sequences in the indicated proteins. A parallel analysis was conducted on pairwise alignments of A1AT with HSPs, and of A1AT and HSPs with the indicated islet proteins, to determine the sequence similarity by means of T-Coffee and Clustal Omega alignment tools (see the Methods section). On the left, the steps leading to the identification of A1AT sequences similar to immunogenic peptides/epitopes identified thus far in islet proteins. On the right, the steps by which further A1AT sequences were discovered similar to islet peptides/epitopes through cross-similarity to the sequences of HSPs similar to islet peptides/epitopes. Some of these A1AT sequences also coincide with those found in the third step on the left, detected through the alignment of A1AT with HSPs (dotted line between the two steps). The convergence point of the two analyses is represented by all the A1AT and HSP sequences with similarity to each other and to islet immunogenic peptides/epitopes. Among these sequences are those identified with higher probability to represent new antigenic peptides in T1D.

In the first step (Fig. 1), A1AT was aligned with any of the islet proteins considered a main autoantigen in T1D, namely, pro-insulin (INS), GAD65, IA-2, ICA69, IAPP and HSP60, and with Grp94 and HSP70, the two HSPs found in complexes with mA1AT in the plasma of diabetic patients^19,20^. HSP60 was included in the analysis since, similarly to other main islet protein antigens, it represented a source of immunogenic peptides, also used as vaccine in human diabetes^22^. The sequences of A1AT with strong similarity to both islet proteins and HSPs were analysed using the msa programme of T-Coffee to test the overall score of cross-similarity. In parallel, Grp94, HSP70 and HSP60 were aligned with INS, GAD65, IA-2, ICA69 and IAPP to detect similar sequences (Fig. 1). The analysis was then centred on the sequences of both A1AT and HSPs with similarity to all the peptides/epitopes of INS, GAD65, ICA69, IA-2 and IAPP reported in the literature as immunogenic in human diabetes. By taking into consideration the sequences of HSPs similar to both islet peptides/epitopes and A1AT, it was possible to identify other sequences in A1AT with cross-similarity to islet epitopes besides those recorded in pairwise alignment with any islet protein (Fig. 1). All of the sequences of A1AT similar to both HSPs and islet protein epitopes were listed, and those with the highest similarity score (100%) to two or more islet protein epitopes were analysed using T-Coffee (https://www.ebi.ac.uk/Tools/msa/tcoffee) and Clustal Omega (https://www.ebi.ac.uk/Tools/msa/clustalo/) which also provide information about the evolutionary relationship of the protein sequence.

To further prove the specificity of sequence similarity, pairwise and msa with the islet proteins and HSPs were also performed with the sequences of human serum albumin (HSA, P02768), bovine serum albumin (BSA, P02769) and vitamin D binding protein (VTDB, P02774) taken as proteins of reference.

## Results

### Sequence similarity between A1AT and islet proteins encompasses similarity to HSPs

**Figure 2:**
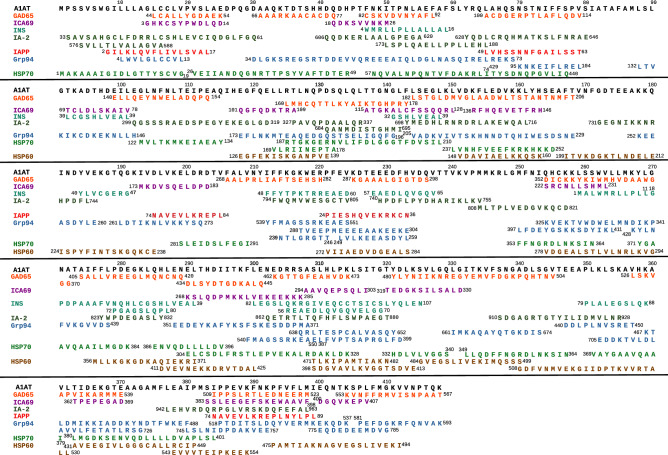
Overview of the sequences of both antigenic islet proteins and HSPs similar to A1AT. Any single protein was aligned with A1AT using the alignment programmes reported in the Methods section and the sequences of at least eight amino acids with similarity to A1AT are listed below the corresponding sequences of A1AT. More than one similar sequence of pro-insulin (INS), IA-2, Grp94, HSP70 and HSP60 is found in correspondence of some sequences of A1AT because of the two distinct alignment programmes that identify separate similar sequences in the same protein.

Among the proteins considered in the alignment with A1AT (Fig. 2), Grp94 showed the most extensive similarity (Tables S1 and S2), whereas fewer similar sequences were found in both HSP70, in which similar sequences were mostly found in the first 440 residues, and HSP60, in which similarity regarded the sequences 398–413 and 431–449 of HSP60 (Fig. 2; Tables S1 and S2) identified as immunogenic peptides^22^.

**Figure 3:**
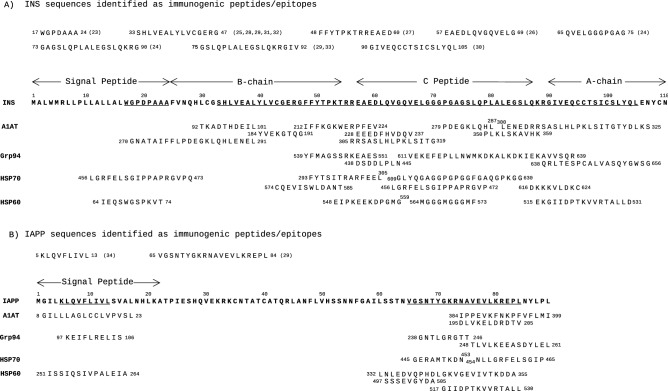
Sequences of A1AT, Grp94, HSP70 and HSP60 similar to immunogenic peptides of INS and IAPP. The sequences of INS (**A**) and IAPP (**B**) reported in the literature as candidate immunogenic peptides/epitopes (references in parenthesis) are listed above and underlined in the corresponding sequence of both INS (**A**) and IAPP (**B**). Similar sequences of A1AT, Grp94, HSP70 and HSP60 are aligned below the sequences of INS and IAPP. In A1AT and HSP70, the same sequence (or part of it) can be found aligned with distinct peptides of INS because of the two methods of alignment that recognise different regions of similarity. The overlapping sequences of A1AT, Grp94 and HSP70 with similarity to the same sequence of INS and IAPP also show a high similarity score when analysed together in msa of T-Coffee. None of the sequences of HSP60 considered immunogenic in T1D satisfied the criteria of similarity to both INS and IAPP.

The islet proteins with extensive similarity to A1AT were INS, GAD65 and IA-2 (Fig. 2; Tables S1 and S2). Almost the entire INS sequence (105 of 110 residues), including all of the known epitopes of INS^23–33^ (Fig. 3A), showed similarity to A1AT (Table 1). In particular, the INS sequences 18–39, 48–70 and 72–107 were similar to distinct A1AT sequences, with the longer ones being A1AT 270–291 and 300–325 (Fig. 2, 3A). Unlike INS, IAPP did not show any extensive sequence similarity to A1AT (Fig. 2; Table S1), although three separate A1AT sequences showed similarity to the immunogenic peptides of IAPP^29,34^ (Fig. 3B).

**Table 1:** Sequences of INS with similarity to sequences of both A1AT and HSPs.

INS (pro-insulin sequence)	A1AT	Grp94	HSP70	HSP60
1–24	47–59/259–269	116–130	164–178/456–473	64–74
18–39	270–291/92–101/141–148	495–512	221–231	482–494
40–61	184–191/212–224	539–551	574–585/293–305	
53–70	305–319/228–237	438–445		548–559
63–85		611-630^a^	456–472/609–630	564–573
70–95	279–287/350–359	614–639 ^a^		
82–107	300–325	627-639^a^/644–656	616–624	515–531

Indicated are all the A1AT and HSP sequences with similarity to INS found in the pair-wise alignment of both A1AT and any HSP with INS. Some A1AT and HSP sequences span two or three sequence intervals of INS. Underlined are the sequences reported in Fig. 3 that encompass the INS peptides recognized as epitopes in T1D.

^a^The sequence 611–639 of Grp94 (see Fig. 3) is similar to the 66–94 sequence of INS that includes the most part of C-peptide. Both T-Coffee and Clustal Omega methods supported the similarity of the 617–656 sequence of Grp94 to INS 72–107.

**Figure 4:**
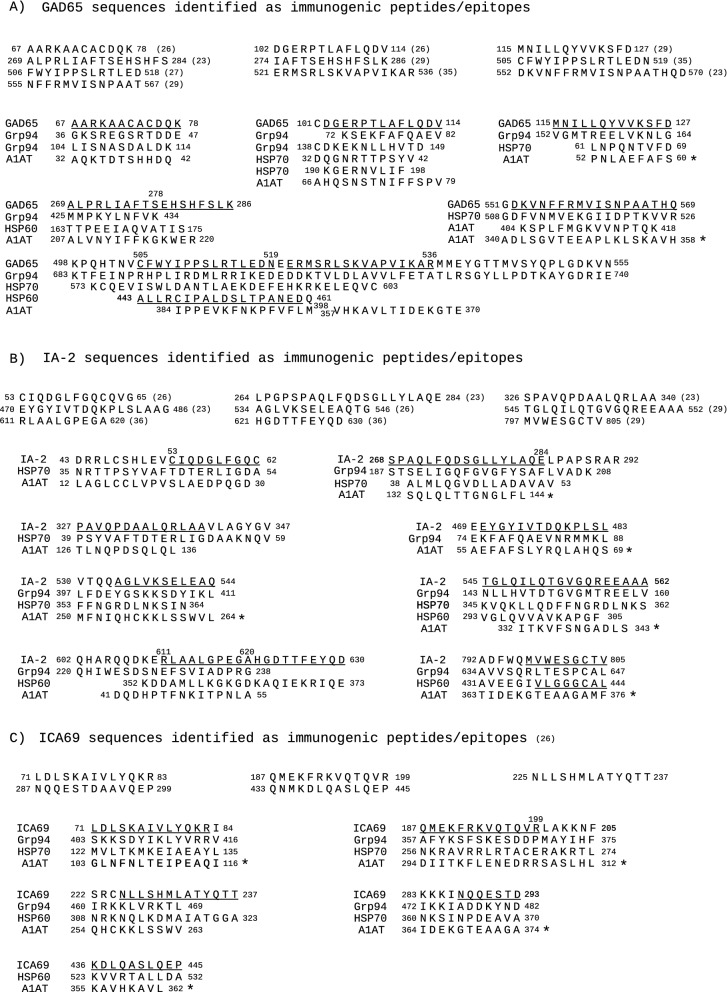
Sequences of Grp94, HSP70 and HSP60 similar to immunogenic peptides/epitopes of GAD65, IA-2 and ICA69. The sequences of GAD65, IA-2 and ICA69 reported in the literature as immunogenic peptides/epitopes (related references in parenthesis) are listed in the upper part of panels (**A**), (**B**) and (**C**), respectively. Pairwise alignment of each HSP with GAD65, IA-2 and ICA69 was made as described (see the Methods section and Fig. 1), and all similar sequences were considered. The figure shows only the sequences of Grp94, HSP70 and HSP60 that are either partly or entirely similar to the immunogenic peptides (underlined) of GAD65 (**A**), IA-2 (**B**) and ICA69 (**C**). In (**A**), multiple sequences of Grp94 and HSP70 are found similar to some immunogenic peptides of GAD65. Both T-Coffee and Clustal Omega identified the long 683–740 sequence of Grp94 similar to GAD65 498–555. Similar to GAD65 505–523 is also the HSP60 sequence 443–461 (underlined) that is part of the 437–460 immunogenic peptide p277^22^. These GAD65 and HSP60 sequences, together with the sequences of overlapping length of Grp94 (690–708) and HSP70 (578–596) are highly similar to each other when analysed in msa of T-Coffee. In (**A**–**C**), below the sequences of HSPs similar to the indicated peptides of each islet protein, are also sequences of A1AT similar to the same islet peptides in pairwise alignment of A1AT with GAD65, IA-2 and ICA69 (Fig. 2). Marked with an asterisk are instead the A1AT sequences that, thanks to the similarity to the indicated HSP sequences, show cross-similarity to the islet peptides in msa and are thus considered as additional sequences in data analysis (Fig. 1; Table 2). Significant similarity was found with msa of T-Coffee for all other groups of sequences of approximately the same length reported in (**A**–**C**).

Of relevance was the broad similarity shared by A1AT with GAD65, because it comprised almost half of the GAD65 residues (Table S2) with very long sequences (Fig. 2), some of which covered the majority of the known GAD65 epitopes^23,26,27,29,35^, including those in the 473–543 immune-dominant peptide^35^ (Fig. S1, Fig. 4A). Importantly, most of the GAD65 sequences similar to A1AT were detected by both alignment methods (Fig. 2).

In addition, in the case of IA-2, there was agreement among the methods in identifying the sequences of IA-2 similar to A1AT as those prevalently located in the cytosolic domain (601–979). Only T-Coffee found four additional similar sequences in the luminal portion of IA-2 (35–575) and another single sequence in the trans-membrane fragment (576–600) (Fig. 2; Table S1). As a result, very long portions of IA-2, mainly in the luminal and trans-membrane domains, were not aligned at all with A1AT, with 276 of 979 residues being thus similar to A1AT (Table S2).

Not so significant was the similarity shared by A1AT with ICA69 (Fig. 2; Table S2) although in this case, the similarity also comprised the epitopes of ICA69 predicted to bind to HLA molecules, (i.e., sequences 69–78 and 222–231; Table S1)^26^.

The overview of the alignments of any protein with A1AT (Fig. 2) demonstrated that many sequences of islet proteins shared with HSPs a similarity to the same sequences of A1AT. Thus, to test in msa the extent of cross-similarity, we grouped the sequences of A1AT similar to at least two other proteins of approximately the same length (Fig. S1). Most A1AT sequences (17 of 25) showed significant cross-similarity to Grp94, either alone or with HSP70 or HSP60, and, in decreasing order of frequency, to GAD65, IA-2, INS, ICA69 and IAPP (Fig. S1). Of interest was the long 144–168 sequence of A1AT with similarity to GAD65 182–206, IA-2 699–716 and Grp94 205–229 (Fig. S1). On the whole, the sequences of A1AT with similarity to the higher number of islet proteins and HSPs were around amino acid (a.a.) 300–320 and 380–400 (Fig. 2, Fig. S1).

### Similarity of HSPs to islet proteins supports broadening of cross-reactivity to A1AT

The finding that many sequences of the islet proteins shared with HSPs, especially Grp94, similarity to A1AT (Fig. 2; Fig. S1) allowed us to hypothesise a broader similarity between HSPs and islet proteins. We investigated this issue by aligning each HSP with islet proteins and recording, among the numerous similar sequences of HSPs (not shown), those with similarity to the immunogenic peptides/epitopes of any islet protein (Fig. 1, steps on right).

As observed with A1AT, also in the alignment with HSPs the sequence of INS was almost entirely covered by similar sequences of Grp94, HSP70 and HSP60 (Table 1). Excluding the 17–24 epitope in the signal peptide, similar to a single sequence of HSP70 and HSP60 (Fig. 3A), the remaining INS sequence, comprising C-peptide and A-chain, showed similarity especially to Grp94 and HSP70. Of note was the long 611–656 sequence of Grp94 with similarity to the 66–107 sequence of INS, identified by both the alignment methods as two separate, partly overlapping peptides (Fig. 3A).

In the pairwise alignment with IAPP, both Grp94 and HSP60 contained sequences similar to almost the entire IAPP sequence (data not shown), including the known epitopes^29,34^ (Fig. 3B), whereas only two single sequences of HSP70 were found similar to IAPP in the portion comprising the 65–84 immunogenic peptide^29^ (Fig. 3B).

In the alignment of HSPs with GAD65, Grp94 showed similarity to all known GAD65 epitopes, with the only exception of the peptide 552–570^23^ similar to HSP70 (Fig. 4A). Of particular relevance was the long 683–740 Grp94 sequence similar to GAD65 498–555, because part of this sequence that comprised two GAD65 epitopes, also showed similarity to HSP70 573–603 and to the 443–461 immunogenic peptide of HSP60^22^ (Fig. 4A). The sequences of Grp94, HSP70 and HSP60 similar to GAD65 epitopes also displayed cross-similarity to the sequence 384–398 of A1AT already found similar to the same GAD65 peptide 509–523 in the alignment of A1AT with GAD65 (Fig. 2, Fig. S1) and to IAPP sequence 74–88 (Fig. S1). The sequences of A1AT similar to those of HSPs with similarity to islet epitopes turned out to display cross-similarity to the same islet epitopes when analyzed in msa (Fig. 4, asterisks on A1AT).

Also in the alignment with IA-2 most of the numerous similar sequences found in HSPs belonged to Grp94, whose sequence covered six of the nine IA-2 epitopes^23,26,29,36^ (Fig. 4B). Thus, the structural similarity to the epitopes of IA-2 was apparently better sustained by Grp94 and HSP70 than by A1AT (Table S1).

Both alignment methods detected an extensive sequence similarity of each HSP with ICA69 (data not shown) including all the sequences identified as immunogenic (Fig. 4C)^26^. This result differed from that obtained with A1AT, which showed limited similarity to ICA69 epitopes (Table S1). However, cross-similarity to ICA69 epitopes was displayed by the A1AT sequences highly similar to HSPs (Fig. 4C, asterisks on A1AT).

To exclude that the broad structural similarity displayed by Grp94 and HSP70 with islet protein epitopes was not specific, i.e., it could also be shared by other HSPs, we extended the investigation to the HSPs with extensive structural similarity to Grp94 and HSP70. Neither HSP90 (80% sequence similarity with Grp94) nor Grp78 (90% sequence similarity with HSP70) showed any sequences with similarity to the epitopes of GAD65 and ICA69, whereas in the pair-wise alignment with IA-2, only a single sequence in HSP90 (505–518), but none in Grp78, was found similar to a single epitope of IA-2 (792–805). An even more impressive difference, with respect to what observed with Grp94 and HSP70, was noted in the alignment of HSP90 and Grp78 with INS, since only two short and distant sequences in INS were covered by similar sequences of both HSP90 and Grp78 (data not shown).

### Sequences of A1AT and HSPs with the best score of similarity to different islet epitopes

**Figure 5:**
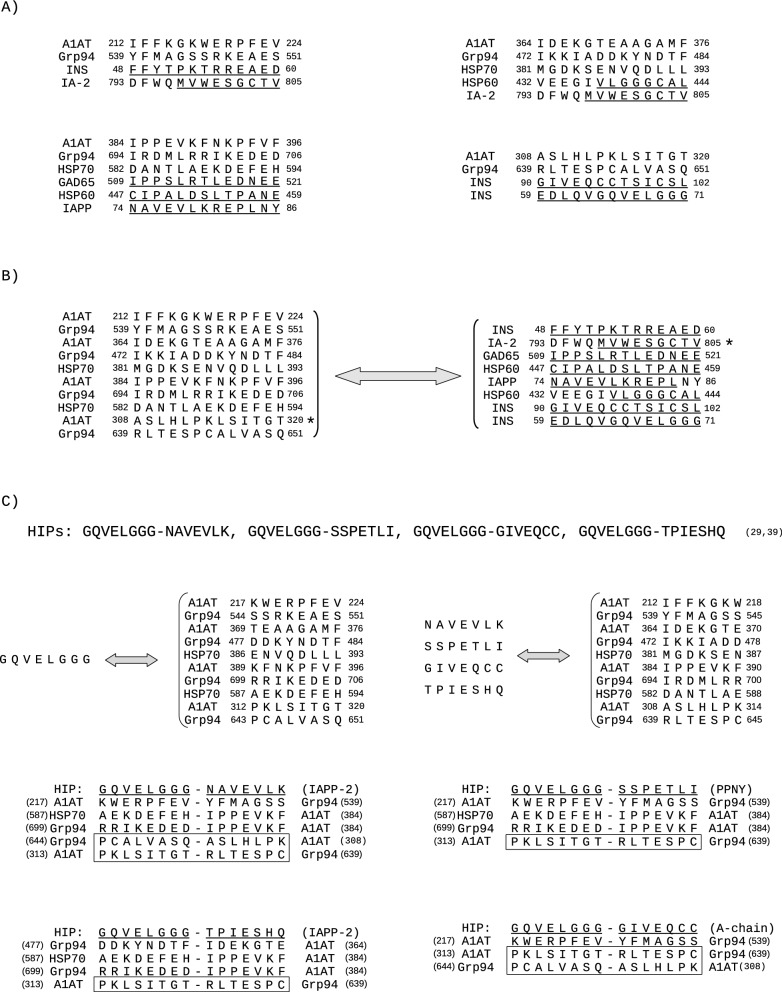
Sequences of A1AT, GRP94 and HSP70 that might better serve as new immunogenic peptides in T1D. (**A**) The four groups of A1AT, Grp94 and HSP70 sequences are chosen from those listed in Table 2 for satisfying the criteria of similarity to at least two islet protein epitopes (underlined in each group) and two HSP sequences. To accurately compare the groups of sequences, the criteria were fixed such that the sequences of each group were perfectly aligned with the maximum score of similarity (100%). Thus, the sequences 293–304 of HSP70, 283–293 of ICA69 and 398–409 of HSP60 were excluded, respectively, from the groups of A1AT 212–224, A1AT 364–376 and A1AT 308–320 (see Table 2) for not contributing to reach the highest score of similarity. (**B**) The groups of sequences as in (**A**) were analysed in msa of T-Coffee. The four sequences of A1AT taken both separately (not shown), and together with the corresponding HSP sequences were found to be highly similar to each other (groups of sequences on left). In addition, when tested together, the islet epitopes originally similar to distinct A1AT and HSP sequences (as in A) turned out to be highly similar to each other (group of sequences on right). Each group of A1AT and HSP sequences as in (**A**), were also tested individually in msa of T-Coffee for similarity to the islet epitopes combined together. All groups, with the exception of A1AT 308–320 gave the maximum score of similarity with all the islet epitopes. When the IA-2 epitope 793–805 was excluded from the analysis, A1AT 308–320 with Grp94 639–651 also yielded the maximum score of similarity to all other islet epitopes when tested both individually and together with all the other groups. The asterisk marks the sequences that in combination slightly reduce the overall score of similarity. (**C**) Hybrid peptides (HIPs) identified as new antigens in T1D (references in parenthesis). The eight right-sided residues of each A1AT and HSP sequence, as in (**B**), were tested in msa with the left common part of each HIP (GQVELGGG) and found to yield the maximum score of similarity (on the left). The same was true for the first seven left-sided residues of each A1AT and HSP sequences as in (**B**), which both singularly and in association, were highly similar (100%) to the four different peptides that form the right part of each HIP (on the right). The two peptides in which each A1AT and HSP sequence was split (the two central residues being common to both peptides) were combined in such a way that an A1AT or HSP sequence similar to the left part of HIP was associated with an A1AT or HSP sequence similar to the right part of any HIP and *vice-versa*. Then, for any A1AT/HSP fused peptide, the extent of similarity to any HIP was evaluated in pairwise alignment with T-Coffee and the peptides that resulted in the maximum similarity score were recorded. The fused A1AT/HSP peptides with similarity to any HIP were also analysed together in msa of T-Coffee to establish the cross-similarity score. In (**C**) are the A1AT/HSP peptides that both in pairwise alignment and in msa gave the maximum score of similarity. Boxed sequences are those with particular relevance as immunogenic peptides.

To identify the sequences of both A1AT and HSPs that might better satisfy the requisites of potential epitopes in diabetes, we considered only those with similarity to islet protein epitopes (Table 2). Twenty-six sequences of A1AT together with a higher number of Grp94 sequences, among those of HSPs, shared mutual similarity to the indicated epitopes of IA-2, INS, GAD65, IAPP and ICA69 (Table 2). As expected from the cross-alignments of both A1AT and HSPs with islet proteins, and of A1AT with HSPs (Figs. 2, 3, 4), the same A1AT sequence showed similarity to distinct islet epitopes and to distinct HSP sequences (Table 2). We chose the sequences of A1AT that, in addition to adequately matching the length of the corresponding islet epitopes, also showed cross-similarity to at least two HSPs and two islet epitopes. The sequences were: A1AT 212–224, 305–321, 363–376 and 384–398 (Table 2). These groups of sequences were analysed in depth after the best alignment with the perfect score of similarity was guaranteed in each group. To this aim, the sequences of HSP70 293–304, ICA69 283–293 and HSP60 398–409 were omitted from the analysis, as they did not support the required extent of similarity and alignment in the group to which they belonged (Fig. 5A). In msa, the four A1AT sequences, considered both alone and together with the corresponding similar sequences of Grp94 and HSP70, were perfectly similar to each other (Fig. 5b, left), implying that each group of sequences could be equally similar to all islet epitopes. This was also confirmed by the unexpected finding that all islet epitopes were highly similar to each other (Fig. 5B, right), suggesting a common structural denominator of both these islet epitopes and the sequences of A1AT, Grp94 and HSP70.

**Table 2:** Sequences of A1AT, Grp94, HSP70 and HSP60 with mutual similarity to immunogenic islet peptides/epitopes.

Grp94	HSP70	HSP60	Epitopes of islet proteins	A1AT
	8–19/35–54		IAPP 2–17/IA-2 39–61	8–30
35–46/104–114	36–47		GAD65 66–78	31–42
45–59/220–238		352–363	IA-2 606–620	41–55
152–164	61–69		GAD65 115–127	52–60
74–88			IA-2 469–483	55–69
138–149	32–42/429–440		GAD65 101–114	66–79
136–145			INS 30–39	92–101
403–415	122–134		ICA69 71–83	103–115
181–191	39–49/188–198		IA-2 327–337	126–136
187–203		38–52	IA-2 268–284	132–144
248–256		346–355/520–530	IAPP 74–84	195–205
425–434		163–175	GAD65 269–284	207–220
539–551	**293–304**		**INS 48–60**/**IA-2 794–805**	**212–224**
397–411	353–364		IA-2 530–544	250–264
325–334/460–469		278–287/308–317	ICA69 222–231	254–263
617–625	617–625	359–367	INS 72–80	279–287
357–375	256–274		ICA69 187–205	294–312
638–652		**398-409**^a^	**INS 56–70**/**INS 87–103**	**305–321**
143–160	345–362	293–305	IA-2 545–562	332–343
		508–526	GAD65 551–569	340–358
624–633	369–377	518–527	INS 79–88	350–359
		523–532	ICA69 436–445	355–362
710–723			GAD65 526–539	357–370
471–488/634–647	**380–393**/**360–370**	**431-444**^a^	**IA-2 792–805**/**ICA69 283–293**	**363–376**
518–531/694–708	**582–596**	**447-461**^a^	**GAD 509–523**/**IAPP 74–88**	**384-398**^a^
581–593		486–494	GAD 553–567	404–418

Sequences of A1AT and HSPs are those found similar to the immunogenic islet peptides/epitopes following the pairwise alignment of both A1AT and each of the HSPs with islet proteins (Figs. 2–4). Underlined HSP sequences are those similar to islet peptides following the alignment of each HSP with islet proteins (Figs. 3, 4), whereas not underlined are those HSP sequences similar to A1AT in the alignment of A1AT with any protein, including HSPs (Fig. 2). Underlined sequences of A1AT are those similar to islet peptides through cross-homology to HSPs (Figs. 1, 4), whereas not underlined are sequences of A1AT derived from the alignment of A1AT with both islet proteins and HSPs (Fig. 2). All sequences in each row show high similarity consensus in msa. In bold are the sequences analysed separately (Fig. 5), because they are similar to more than one islet peptide/epitope and HSP.

^a^HSP60 sequences identified as immunogenic peptides; the 431–461 sequence includes the 437–460 p277peptide also used as a vaccine in T1D.

Looking deeper into how these A1AT and HSP sequences could be related to each other in supporting similarity to the islet epitopes, any single group, as well as all the four groups of A1AT and HSP sequences, were analysed together with all islet epitopes in msa, and the score of similarity and evolutionary relationship were recorded (Fig. 5B). The groups of A1AT and HSP sequences considered together were perfectly aligned to all islet epitopes with the maximal similarity score reached after excluding the IA-2 epitope. This epitope was perfectly aligned with A1AT 212–224, 364–376 and 384–398, but not equally well with A1AT 308–320 (Fig. 5B). The evolutionary analysis of the sequences indicated that A1AT 308–320 and Grp94 639–651 were, respectively, closely associated with INS 90–102 and 59–71 and had a recent common ancestor (data not shown). It is worth noting that none of the reference proteins was able to support the similarity to all islet epitopes as did the above sequences of A1AT and HSPs.

To ascertain whether other islet proteins related to pathogenesis of human diabetes could support structural similarity to A1AT and HSPs as did the main islet protein epitopes, we considered the epitopes identified in ZnT8^26,37^, IGRP^38^, UCN3^37^, ISL-1^37^ as well as the immunogenic peptide (DRiP) derived from an alternative open reading frame within the human insulin mRNA with the capacity to sustain autoimmunity in clinical diabetes^39^. Whereas no peptide, either epitope or not, of the above islet proteins showed similarity to any of the above sequences of A1AT, Grp94 and HSP70 (Figs. 4, 5A), a high score of cross-similarity to some of these A1AT and HSP sequences was instead displayed by DRiP due to its main similarity to INS 1–10 (Fig. S2). Since DRiP was also found responsible for killing beta cells^39^, the structural similarity to the INS-like epitope appears of particular relevance for driving antigenicity in human diabetes.

### Sequences of A1AT and HSPs that better satisfy the requisite of antigenic peptides in T1D

In consideration of the above results, we extended the investigation also to the recently identified neo-antigens, hybrid insulin peptides (HIPs), that are products of the post-translational modification of islet proteins^40,41^. HIPs are formed with part of the C-peptide fused with either pancreatic neuropeptide Y (NNPY), IAPP or A-chain and are key antigens for auto-reactive T cells in T1D^29,42^. We tested whether the HIPs that activate human CD4 T cells showed similarity to the sequences of both A1AT and HSPs with cross-similarity to islet epitopes (Fig. 5B). We noted that HIPs, regardless of whether their right part was from NNPY, IAPP or A-chain, were perfectly similar to each other and that the sequences of A1AT, Grp94 and HSP70 similar to all the islet peptides (Fig. 5B) had the first seven residues similar to the peptides of the right part in HIPs and the last eight residues similar to the common (GQVELGGG) peptide of each HIP, the two peptides of each A1AT and HSP sequence having the two central residues in common (Fig. 5C). We thus tested in pairwise alignment and in msa the extent of similarity between each HIP and the peptides obtained by fusing the sequences of A1AT with those of either Grp94 or HSP70, with each sequence being similar to either the right or left part of any HIP, so as to satisfy all the possible combinations in each group of A1AT and HSP sequences (Fig. 5C). The A1AT/HSP peptides that best aligned with HIPs with the highest score of similarity also in msa were from A1AT 384–396 with Grp94 694–704 and from A1AT 308–320 with Grp94 639–651 (Fig. 5C). This Grp94 sequence was also evolutionarily closely related to INS 90–102 (data not shown). Only these last A1AT and Grp94 sequences contained the peptides that in different combinations were perfectly similar to all HIPs (Fig. 5C).

## Discussion

**Figure 6:**
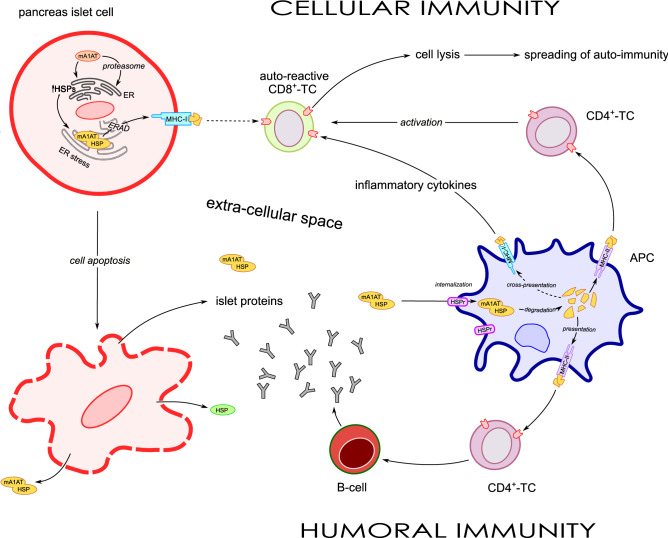
Schematic representation of the mechanisms by which mA1AT might trigger autoimmunity in T1D. The expression of mA1AT in the plasma of diabetic subjects was of variable entity but much lower than that of wA1AT^19^, so that both intensity and time of appearance of autoimmune reactions are expected to vary largely among affected persons. Following the appearance of mA1AT in the cell a first intervention of cytoplasm HSPs (HSP70) is predicted to take place to chaperone the protein before the entry into the ER (a preliminary passage through proteasome might also be possible). In the ER, mA1AT triggers UPR with stimulation of ER-resident HSPs recruited to correct the unfolded protein. The unsuccessful attempt makes mA1AT become a substrate of ER-associated degradation with the main assistance of Grp94^17,44^. Degradation products of mA1AT in complex with HSPs (Grp94) form the primary antigen (Ag) loaded onto MHC-class I molecules of islet cells for presentation to CD8^+^ T cells (TCs). Although activation of cellular immunity can also occur at this stage (dashed line), the more probable outcome of prolonged ER stress is cell apoptosis^16^ with extracellular liberation of Ag (soluble Ag) and HSPs, responsible for the activation of humoral immunity. This mechanism predicts the production of Abs initially directed against the immunogenic component of Ag (i.e., HSP), but also rapidly targeting A1AT and the islet proteins with structural similarity to HSPs. In the absence of other accelerating factors (inflammation, cross-reactive Ags), the activation of humoral immunity, documented by circulating Abs against the main islet proteins, can remain unnoticed for a long time without any clinical manifestation of disease (silent diabetes). The soluble Ag is captured by HSP receptors (HSPr) on APCs^52^ and, after internalisation and degradation in the endocytic and phagocytic pathways, loaded onto MHC-class II molecules for activation of CD4^+^ TCs (the main pathway of Ag presentation) and on MHC-class I molecules (cross-presentation of the Ag) for activation of CD8^+^ TCs (cellular immunity). Activated CD4^+^ TCs in turn further activate auto-reactive CD8^+^ TCs with lysis of islet cells and production of inflammatory cytokines that also induce over-expression of MHC-class I molecules on islet cells. The spreading of cross-reactive Ags characterises the stage of propagation and intensification of autoimmune processes in which the cellular arm of immunity marks the clinical feature of overt diabetes.

Based on previous results indicating that complexes of mA1AT with HSP70 and Grp94 found in the plasma of type 1 diabetic subjects^20,43^ might be markers of disease with potential immunogenic relevance for disease development, we explored here the possibility that A1AT and HSPs display structural similarity to the peptides already identified in islet proteins as immunogenic in human diabetes. If the assumption is true that mA1AT is the very first trigger of intracellular events causally related to HSP association and extracellular liberation^16,17,44^ (Fig. 6), identifying the peptides that in the complex have strong structural similarity to already known antigens in diabetes can help to prove the immunogenic potential of these new peptides. Thus, although the in silico analysis is preliminary and partial, it might be useful for providing the necessary indications.

Our results first demonstrate a significant, broad sequence similarity between A1AT and Grp94 (Fig. 2; Table S1), which likely predicts the spreading to A1AT of the reactivity arising against the HSP. In fact, HSPs outside the cell are highly immunogenic, and in particular Grp94 are found in the plasma of diabetic patients, even years after diabetes onset^20^. Thus, HSPs could be primarily responsible for any autoimmune reaction triggered by the complex, also demonstrated by the fact that anti-HSP Abs circulate in the plasma of diabetic patients^42^. Cross-reactivity of anti-HSP Abs with many structurally similar portions of A1AT can also account for the reduced plasma concentration and defective functioning of A1AT frequently observed in T1D^45,46^.

Implication of the mA1AT/HSP complex in the autoimmune process related to diabetes is supported by the significant structural similarity shared by both HSPs, Grp94 in particular, and A1AT with all the peptides/epitopes already identified in the major islet antigen proteins (i.e., INS, GAD65 and IA-2; Figs. 3, 4), a condition not satisfied instead by any of the reference proteins nor by any of the HSPs that show a high degree of structural similarity with Grp94 and HSP70. Particularly striking was the result of almost complete coverage of the INS sequence by distinct similar sequences of A1AT and by very long sequences of Grp94 (Table 1; Fig. 3A). The possibility that the structural similarity shared by A1AT and Grp94 with INS also implies a functional link is supported by the fact that the A1AT expression is regulated by hepatocyte nuclear factors (HNFs) which are involved in the regulation of many genes in human islets^47^, and that some *HNF* variants are related to the development of monogenic forms of diabetes^48^. Further evidence also comes from the functional link that exists between the A1AT expression and a variant form of *HNF1A* associated with impaired insulin secretion^14^ and by the demonstration that Grp94 has an essential role in regulating beta cell function and pro-insulin level in human beta cells^49^.

In line with these considerations are the results of the alignments of A1AT and HSPs with GAD65 and IA-2 (Fig. 4). More than 60% of A1AT residues displayed similarity with both GAD65 and IA-2 (Table S2) in long sequences (≥ 15 residues), whereas, among HSPs, Grp94 contained a higher number of sequences and residues (450) similar to GAD65. Of particular interest was the Grp94 sequence of 58 residues covering two separate epitopes of GAD65, also similar to two separate sequences of A1AT (Fig. 4A). In the case of IA-2, similarities with A1AT were found almost always exclusively in the cytoplasmic segment of IA-2 which undergoes cleavage and translocation to the nucleus where it enhances pancreatic beta cell proliferation and promotes the expression of insulin genes^50^. The similarity of A1AT with the cytoplasmic IA-2 fragment further suggests A1AT’s functional involvement in the pathway that links insulin secretion with gene expression in beta cells.

Similar considerations can be applied to the extensive sequence similarity found between IA-2 and HSPs, characterised by the remarkable length of some sequences (> 40 residues) distributed rather homogeneously along the entire IA-2 sequence. Compared with HSP70 and HSP60, Grp94 again displayed a greater number of similar residues (486) prevalently covering the trans-membrane and cytoplasmic fragments of IA-2 with very long sequences. A recent study proposed that autoimmunity triggered by GAD65 and IA-2 is linked to the release of these proteins in exosomes in association with ER chaperones, including Grp94^51^. The conclusion that immunogenicity is primarily linked to the HSPs leaving the ER under stress conditions corroborates our results that indicate that a broad structural similarity can justify the spreading of autoimmunity from HSPs to other islet proteins (Fig. 6).

Because many sequences of A1AT and HSPs showed similarity to the major islet antigens (Fig. S1; Table 2), we identified the sequences that might best constitute immunogenic peptides. Among all A1AT sequences partly overlapping each other in covering separate islet epitopes, we considered only those with similarity to at least two islet epitopes and HSPs; intriguingly, these A1AT sequences were highly similar to each other and the corresponding Grp94 and HSP70 sequences (Fig. 5B). Since also the epitopes of INS, GAD65, IA-2, IAPP and HSP60 were highly similar to each other (Fig. 5B) it followed that the A1AT and HSP sequences, taken both separately and together, shared similarity with all the islet epitopes (Fig. 5B), a finding suggesting that a common structural denominator was shared by A1AT and HSPs with these islet epitopes. We verified whether this conclusion could be extended to other autoantigens of islet proteins, as well as to newly discovered peptides of insulin targeted by reactive T-cells in human diabetes, such as the hybrid insulin products (HIPs)^29,42^ and the defective immunogenic peptide translated from the insulin mRNA^39^. Negative results were obtained by testing the epitopes of ZnT8, IGRP, UCN3 and ISL-1 whereas the insulin-related epitopes, both non-conventional (Fig. S2) and hybrid (Fig. 5C) turned out to display strong similarity to the A1AT/HSP sequences with similarity to the panel of the main islet peptides/epitopes (Fig. 5B). Apparently thus, the structural similarity to specific INS epitopes was crucial for supporting cross-similarity of the A1AT and HSP sequences to other main islet antigens of GAD65 and IA-2. Although sequence similarity does not equate to functional similarity, by investigating protein–protein interaction in String database, we observed that a close association, implying functional relationship, occurred between A1AT and Grp94, but not other HSPs (Fig. S3) and that INS covered a central node that functionally linked two separate clusters of proteins, one comprising A1AT together with the HSPs, the other formed with GAD65 and IA-2 characterized by a closer functional linkage to INS (Fig. S3).

## Supplementary Information

Supplementary Figure 1.

Supplementary Figure 2.

Supplementary Figure 3.

Supplementary Figure 1 Caption.

Supplementary Figure 3 Caption.

Supplementary Figure 2 Caption.

Supplementary Table 1.

Supplementary Table 2.

## Data Availability

All data are available from the authors on request.

## Abbreviations

A1AT: Alpha 1-antitrypsin
a.a.: Amino acid
BSA: Bovine serum albumin
ER: Endoplasmic reticulum
HIP: Hybrid insulin peptides
HSA: Human serum albumin
HSP: Heat Shock Protein
msa: Multiple sequence alignments
UPR: Unfolded protein response
VTDB: Vitamin D binding protein
