# Supplementary material for: A mutant α1antitrypsin in complex with heat shock proteins as the primary antigen in type 1 diabetes in silico investigation
Source: Sci Rep. 2021 Feb 4;11:3002. doi: 10.1038/s41598-021-82730-2 (PMC7862655; doi:10.1038/s41598-021-82730-2)
Supplement: Supplementary file 1 — Supplementary Figure 1. [file 41598_2021_82730_MOESM1_ESM.pdf]

A1AT	8	G I L L L A G L C C L V P V S L A E D P Q G D	30
IA-2	39	G C L F D R R L C S H L E V C I Q D G L F G Q	61
IAPP	2	G I L K L Q V F L I V L S V A L	17
HSP70	8	G I D L G T T Y S C V G	19

A1AT	31	A A Q K T D T S H H D Q	42
GAD65	66	A A R K A A C A C D Q	77
Grp94	35	L G K S R E G S R T D D	46
HSP70	36	R T T P S Y V A F T D T	47

A1AT	46	T F N K I T P N L A E F	57
GAD65	82	C S K V D V N Y A F L	92
INS	4	W M R L L P L L A L L	14
IA-2	611	R L A A L G P E G A	620
Grp94	50	Q R E E E A I Q L D G L	61
HSP70	57	N Q V A L N P Q N T	66

A1AT	65	L A H Q S N S T N I F F S P V	79
GAD65	100	A C D G E R P T L A F L Q D V	114
IA-2	631	L C R Q H M A T K S L F N R A	645
IAPP	50	L V H S S N N F G A I L S S T	63
HSP70	429	T Y S D N Q P G V L I Q	440

A1AT	92	T K A D T H D E I L	101
ICA69	69	T C L D L S K A I V	78
INS	30	L C G S H L V E A L	39
Grp94	136	I K C D K E K N L L	145

A1AT	103	G L N F N L T E I P E	113
GAD65	144	E Y N W E L A D Q P Q	154
IA-2	300	G S S S R A E D S P E	310
HSP70	122	M V L T K M K E I A E	132

A1AT	118	E G F Q E L L R T L N Q P D	131
Grp94	173	E F L N K M T E A Q E D G Q	186
HSP60	126	E G F E K I S K G A N P V E	139

A1AT	125	R T L N Q P D S Q L Q L T T G N G L F	143
GAD65	160	L M H C Q T T L K Y A I K T G H P R Y	178
IA-2	327	P A V Q P D A A L Q R	337
HSP70	187	R T G K G E R N V L I F D L G G G T F	205

A1AT	144	L S E G L K L V D K F L E D V K K L Y H S E A F T	168
GAD65	182	L S T G L D M V G L A A D W L T S T A N T N M F T	206
IA-2	699	M E D H L R N R D R L A K E W Q A L	716
Grp94	205	V A D K V I V T S K H N N D T Q H I W E S D S N E	229

A1AT	151	V D K F L E D V K K L Y H	163
Grp94	212	T S K H N N D T Q H I W E	224
HSP70	238	V N H F V E E F K R K H K	250
HSP60	148	V D A V I A E L K K Q S K	160

A1AT	193	I V D L V K E L D R D	203
ICA69	173	M K D V S Q E L D P D	183
Grp94	264	I K N L V K K Y S Q	273

A1AT	212	I F F K G K W E R P F E V	224
GAD65	274	I A F T S E H S H	282
INS	48	F F Y T P K T R R E A E D	60
IA-2	794	F W Q M V W E S G C T V	805
Grp94	539	Y F M A G S S R K E A E S	551

A1AT	221	P F E V K D T E E E D F H	233
IAPP	24	P I E S H Q V E K R K C N	36
Grp94	292	P M E E E E A A K E E K E	304
HSP60	272	V I I A E D V D G E A L S	284

A1AT	254	Q H C K K L S S W V L L M K Y L G	270
GAD65	352	D I C K K Y K I W M H V D A A W G	368
ICA69	222	S R C N L L S H M L	231
Grp94	325	K V E K T V W D W E L M N D I K P	341
HSP60	278	V D G E A L S T L V L N R L K V G	294

A1AT	274	A I F F L P D E G K L Q H L E N	289
GAD65	405	S A L L V R E E G L M Q N C N Q	420
INS	22	A A A F V N Q H L C G S H L V E	37
HSP60	356	M L L K G K G D K A Q I E K	369
IA-2	823	Y W P D E G A S L Y	832

A1AT	290	E L T H D I I T K F L E	301
GAD65	434	D L S Y D T G D K A L Q	445
ICA69	268	K L S Q D P M K K L V E	279
Grp94	353	D E Y K A F Y K S F S K	364
HSP70	304	E L C S D L F R S T L E	315
HSP60	415	N E K K D R V T D A L	425

A1AT	303	E D R R S A S L H L P K	314
GAD65	462	K G T T G F E A H V D K	473
INS	85	S L Q K R G I V E Q C C	96
IA-2	863	E T R T L T Q F H F L S	874
HSP70	317	V E K A L R D A K L D K	328

A1AT	308	A S L H L P K L S I T G	319
ICA69	294	A A V Q E P S Q L I	303
INS	90	G I V E Q C C T S I C S	101
INS	59	E D L Q V G Q V E L G G	70
IA-2	868	T Q F H F L S W P A E G	879
Grp94	639	R L T E S P C A L V A S	650
HSP60	398	S D G V A V L K V G G	408

A1AT	325	S V L G Q L G I T K V F S N G A	341
GAD65	484	I I K N R E G Y E M V F D G K P	499
HSP60	484	G V E G S L I V E K I M Q S S S	499

A1AT	337	S N G A D L S G V	345
IA-2	910	S D G A G R T G Y	539
HSP70	354	F N G R D L N K S	362

A1AT	350	P L K L S K A V H K A	360
INS	79	P L A L E G S L Q K	88
Grp94	443	P L N V S R E T	450
HSP70	369	V A Y G A A V Q A A	378

A1AT	357	V H K A V L T I D E K G T E	370
GAD65	526	L S K V A P V I K A R M M E	539
Grp94	710	V L D L A V V L F E T A T L	723

A1AT	363	T I D E K G T E A A G A M F L E A I P	381
Grp94	471	M I K K I A D D K Y N D T F W K E F	488
HSP70	380	L M G D K S E N V Q D L L L L D V A P	398
HSP60	431	A V E E G I V L G G G C A L L R C I P	449

A1AT	384	I P P E V K F N K P F V F L M	398
GAD65	509	I P P S L R T L E D N E E R M	523
ICA69	384	S L E E G E F S K E W A A V F	398
IA-2	949	R P G L V R S K D Q F E F A L	963
IAPP	74	N A V E V L K R E P L N Y L P	88
Grp94	518	P T D I T S L D Q Y V E R M	531

A1AT	404	K S P L F M G K V V N P T Q K	418
GAD65	553	K V N F F R M V I S N P A A T	567
Grp94	581	P E F D G K R F Q N V A K	593
HSP60	486	E G S L I V E K I	494
