# Supplementary material for: A mutant α1antitrypsin in complex with heat shock proteins as the primary antigen in type 1 diabetes in silico investigation
Source: Sci Rep. 2021 Feb 4;11:3002. doi: 10.1038/s41598-021-82730-2 (PMC7862655; doi:10.1038/s41598-021-82730-2)
Supplement: Supplementary file 2 — Supplementary Figure 2. [file 41598_2021_82730_MOESM2_ESM.pdf]

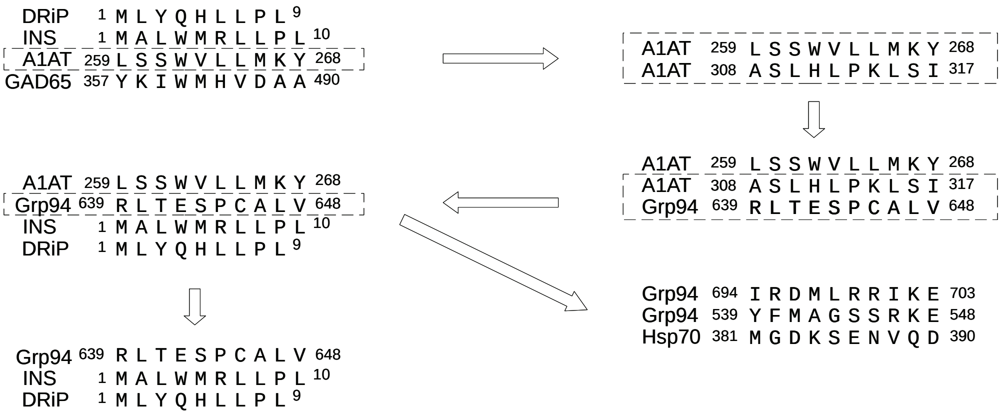
DRiP 1 M L Y Q H L L P L 9
INS 1 M A L W M R L L P L 10
A1AT 259 L S S W V L L M K Y 268
GAD65 357 Y K I W M H V D A A 490
A1AT 259 L S S W V L L M K Y 268
A1AT 308 A S L H L P K L S I 317
A1AT 259 L S S W V L L M K Y 268
A1AT 308 A S L H L P K L S I 317
Grp94 639 R L T E S P C A L V 648
A1AT 259 L S S W V L L M K Y 268
Grp94 639 R L T E S P C A L V 648
INS 1 M A L W M R L L P L 10
DRiP 1 M L Y Q H L L P L 9
Grp94 694 I R D M L R R I K E 703
Grp94 539 Y F M A G S S R K E 548
Hsp70 381 M G D K S E N V Q D 390
Grp94 639 R L T E S P C A L V 648
INS 1 M A L W M R L L P L 10
DRiP 1 M L Y Q H L L P L 9
