# Supplementary figures and images for: A mutant α1antitrypsin in complex with heat shock proteins as the primary antigen in type 1 diabetes in silico investigation
Source: Sci Rep. 2021 Feb 4;11:3002. doi: 10.1038/s41598-021-82730-2 (PMC7862655; doi:10.1038/s41598-021-82730-2)

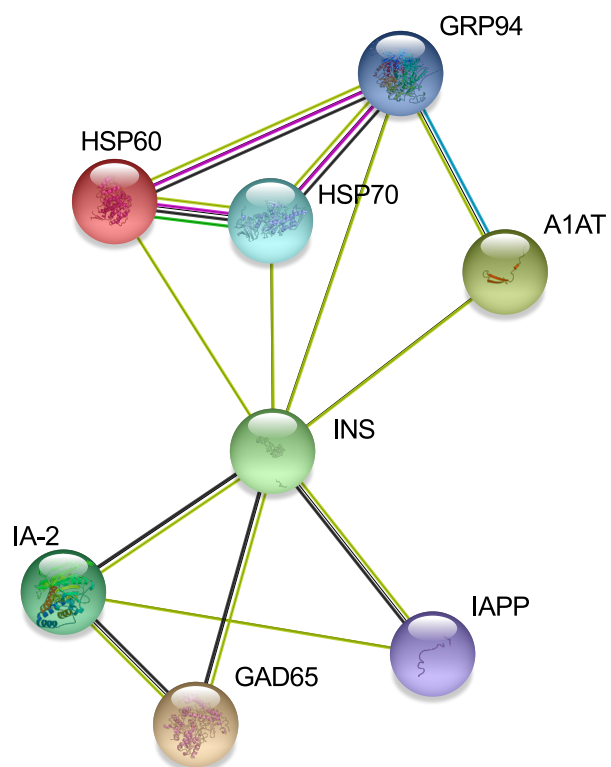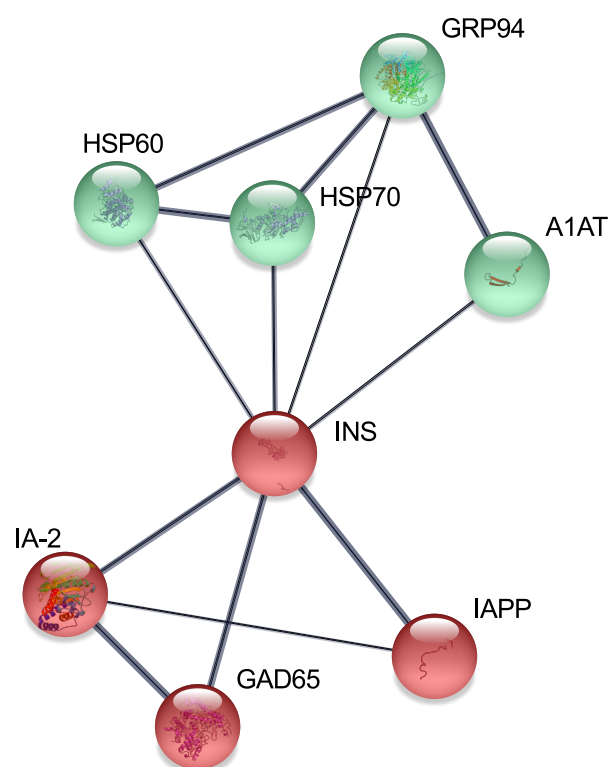

Supplement: Supplementary file 3 — Supplementary Figure 3. [file 41598_2021_82730_MOESM3_ESM.pdf]
