# Supplementary material for: A mutant α1antitrypsin in complex with heat shock proteins as the primary antigen in type 1 diabetes in silico investigation
Source: Sci Rep. 2021 Feb 4;11:3002. doi: 10.1038/s41598-021-82730-2 (PMC7862655; doi:10.1038/s41598-021-82730-2)
Supplement: Supplementary file 4 — Supplementary Figure 1 Caption. [file 41598_2021_82730_MOESM4_ESM.docx]

A mutant α1antitrypsin in complex with heat shock proteins as the primary antigen in type 1 diabetes

*In silico* investigation

Paola Finotti, Andrea Pagetta Dept. Pharmaceutical and Pharmacol Sciences, University of Padua, Italy

**Fig. S1 Sequences of A1AT with crossed similarity with pancreas islet proteins and HSPs**. The sequences of A1AT with similarity to sequences of two or more islet proteins and/or HSPs, as shown in Figure 2, are grouped and similarity tested in msa of T-Coffee. Underlined are the sequences of the islet proteins identified as immunogenic peptides/epitopes in human T1D (see also Figs. 3 and 4). Each group of sequences (also in the case of different length of the sequences) has a high score of similarity.
