# Supplementary material for: A mutant α1antitrypsin in complex with heat shock proteins as the primary antigen in type 1 diabetes in silico investigation
Source: Sci Rep. 2021 Feb 4;11:3002. doi: 10.1038/s41598-021-82730-2 (PMC7862655; doi:10.1038/s41598-021-82730-2)
Supplement: Supplementary file 5 — Supplementary Figure 3 Caption. [file 41598_2021_82730_MOESM5_ESM.docx]

A mutant α1antitrypsin in complex with heat shock proteins as the primary antigen in type 1 diabetes

*In silico* investigation

Paola Finotti, Andrea Pagetta Dept. Pharmaceutical and Pharmacol Sciences, University of Padua, Italy

**Fig. S3. Protein-protein interactions with functional implications.** We searched in String database (<https://string-db.org>) for known and predicted interactions among the proteins investigated as source of peptides of relevance in human diabetes. The resulting network shows significantly more interactions than expected (p<0.0001). Image on the left: coloured edges (associations) among nodes (proteins) indicates the type of interaction evidence (more numerous among HSPs) whereas edge thickness indicates the strength of data support. Associations are meant to be specific and meaningful since joined proteins share common function. The image on the right shows the network represented as two clusters, one comprising INS with GAD65, IA-2 and IAPP, the other comprising HSPs and A1AT. Also when other HSPs (HSP90, Grp78, HSP40) were added in the network, it was apparent that HSPs together with A1AT formed a separate cluster in which each HSP was closely linked to any other HSP, but only Grp94 was also linked to A1AT (data not shown).
