# Supplementary material for: A mutant α1antitrypsin in complex with heat shock proteins as the primary antigen in type 1 diabetes in silico investigation
Source: Sci Rep. 2021 Feb 4;11:3002. doi: 10.1038/s41598-021-82730-2 (PMC7862655; doi:10.1038/s41598-021-82730-2)
Supplement: Supplementary file 6 — Supplementary Figure 2 Caption. [file 41598_2021_82730_MOESM6_ESM.docx]

A mutant α1antitrypsin in complex with heat shock proteins as the primary antigen in type 1 diabetes

*In silico* investigation

Paola Finotti, Andrea Pagetta Dept. Pharmaceutical and Pharmacol Sciences, University of Padua, Italy

**Fig. S2 Sequences of A1AT, Grp94 and HSP70 with similarity to the epitope of a defective ribosomal insulin gene product in T1D.** The epitope of the defective ribosomal insulin product (DRiP), as reported in the paper of Kracht et al.[39], was tested in msa and the best degree of similarity (score 1000) was found with INS 1-10 and the sequences of both A1AT (259-268) and GAD65 (357-366) aligned with INS 1-10 (see Fig. 2). Since A1AT 259-268 was perfectly similar to A1AT 308-317, in turn aligned with Grp94 639-648 (Fig. 2 and Fig. 5A), it turned out that a high score of similarity was also found when A1AT 259-268, INS 1-10, DRiP and Grp94 639-648 were tested together. Other two sequences of Grp94, also located in its C terminus, and one sequence of HSP70 (381-390) were able to replace Grp94 639-648 in supporting a high score of cross-similarity with A1AT, INS and DRiP. However, only Grp94 639-648 maintained a high score of cross-similarity (>92%) when tested alone with INS and Drip.
