# Supplementary material for: A mutant α1antitrypsin in complex with heat shock proteins as the primary antigen in type 1 diabetes in silico investigation
Source: Sci Rep. 2021 Feb 4;11:3002. doi: 10.1038/s41598-021-82730-2 (PMC7862655; doi:10.1038/s41598-021-82730-2)
Supplement: Supplementary file 7 — Supplementary Table 1. [file 41598_2021_82730_MOESM7_ESM.docx]

A mutant α1antitrypsin in complex with heat shock proteins as the primary antigen in type 1 diabetes

*In silico* investigation

Paola Finotti, Andrea Pagetta Dept. Pharmaceutical and Pharmacol Sciences, University of Padua, Italy

INS 1-16 (2) 18-39 (3) 40-47 48-70 (3) 72-107 (3)

(110 a.a.)

GAD65 44-54 66-77 82-92 100-114 140-154 160-178 182-206

(585 a.a.) 268-282 287-298 352-370 405-420 434-445 462-473 480-504

509-523 526-539 553-567

ICA69 3-14 18-26 69-78 101-109 115-128 136-146 173-183

(483 a.a.) 222-231 268-285 294-303 319-330 362-369 383-398 400-407

IA-2 33-61 173-188 299-319 327-337 576-588 606-620 628-646

(979 .aa.) 684-695 698-716 731-755(2) 794-805 808-821 823-832 862-880 910-928 942-963

IAPP 2-17 24-36 49-63 74-89 (2)

(89 a.a.)

Grp94 4-13 34-73 95-104 132-146 173-196 205-229 239-246

(803 a.a.) 249-273 (3) 288-304 325-341 351-371 387-411 (2) 428-439 440-450 467-488 518-537 539-551 (3) 581-593 638-652 661-674 705-726 745-757 775-785

HSP70/1 1-19 26-49 57-74 126-133 169-178 187-210 237-252

(641 a.a.) 281-291 304-328 332-340 349-364 (2) 369-401 (4) 429-440

HSP60 126-139 148-160 199-212 224-238 272-294 (2) 356-371 398-425 (2)

(573 a.a.) 431-449 471-499 (3) 508-530 543-554

**Table S1 Sequences of pancreas islet proteins and HSPs with similarity to A1AT.** Numbers identifying each sequence correspond to the a.a. residues numbered starting from the N terminus of each protein, as reported extensively in Fig. 2. INS stands for pro-insulin. Underlined are the sequences corresponding to or partially including the sequences identified as immunogenic peptides/epitopes in type 1 diabetes. In parentheses is the number of separate A1AT sequences that are similar to the a.a. residues of the indicated sequences
