# Supplementary material for: A mutant α1antitrypsin in complex with heat shock proteins as the primary antigen in type 1 diabetes in silico investigation
Source: Sci Rep. 2021 Feb 4;11:3002. doi: 10.1038/s41598-021-82730-2 (PMC7862655; doi:10.1038/s41598-021-82730-2)
Supplement: Supplementary file 8 — Supplementary Table 2. [file 41598_2021_82730_MOESM8_ESM.docx]

| **A1AT (a.a.)** | **1-90** | **91-180** | **181-270** | **271-360** | **361-418** |  | N° sequences (total a.a) |
| --- | --- | --- | --- | --- | --- | --- | --- |
| GAD65  (585 a.a.) | 4 | 3 | 3 | 4 | 3 |  | **17 (263)** |
| ICA69  (483 a.a.) | 2 | 4 | 2 | 3 | 3 |  | **14 (158)** |
| INS  (110 a.a.) | 1 | 2 | 4 | 5 | - |  | **12 (105)** |
| IA-2  (979 a.a.) | 5 | 5 | 3 | 3 | 1 |  | **17 (276)** |
| IAPP  (89 a.a.) | 2 | - | 2 | - | 1 |  | **5 (60)** |
| Grp94  (803 a.a.) | 3 | 3 | 8 | 8 | 6 |  | **28 (402)** |
| HSP70  (641 a.a.) | 4 | 4 | 2 | 6 | 1 |  | **17 (230)** |
| HSP60  (573 a.a.) | - | 3 | 3 | 6 | 3 |  | **15 (211)** |
| Sum of similar sequences/ | **21** | **24** | **27** | **35** | **18** |  |  |

**Table S2 Number of sequences and amino acid residues of both islet proteins and HSPs with similarity to A1AT at the specified sequence intervals.** For each protein (length in a.a. in parentheses) is indicated the number of sequences similar to the sequence of A1AT considered at intervals of 90 a.a., as reported in Fig. 2. The sequences with similarity at the intersection of each A1AT interval are calculated only once and attributed to the interval containing the higher number of a.a. of that sequence. The overall number of sequences of each protein similar to the entire sequence of A1AT is shown on right, together with the corresponding total number of a.a. residues (taken as the real index of the extent of similarity given the different length of each sequences). The total number of sequences of all proteins similar to A1AT at the specified A1AT interval is reported below

A mutant α1antitrypsin in complex with heat shock proteins as the primary antigen in type 1 diabetes

*In silico* investigation

Paola Finotti, Andrea Pagetta Dept. Pharmaceutical and Pharmacol Sciences, University of Padua, Italy
